# Patterns and Prevalence of Germline *BRCA1* and *BRCA2* Mutations among High-Risk Breast Cancer Patients in Jordan: A Study of 500 Patients

**DOI:** 10.1155/2020/8362179

**Published:** 2020-07-14

**Authors:** Hikmat Abdel-Razeq, Lama Abujamous, Dima Jadaan

**Affiliations:** ^1^Department of Medicine, King Hussein Cancer Center, Amman, Jordan; ^2^School of Medicine, University of Jordan, Amman, Jordan; ^3^Department of Cell Therapy & Applied Genomic, King Hussein Cancer Center, Amman, Jordan

## Abstract

**Purpose:**

Knowledge of *BRCA1* and *BRCA2* mutations has a significant clinical impact on the management and prevention of breast cancer. In this study, we evaluate the pattern and prevalence of germline mutations in *BRCA1* and *BRCA2* among high-risk Jordanian breast cancer patients selected as per international guidelines.

**Methods:**

*BRCA1* and *BRCA2* testing were performed at a reference genetic lab. Mutations were classified as pathogenic/likely pathogenic and variant of uncertain significance (VUS).

**Results:**

A total of 517 patients, median age: 39 (range: 19–78) years, were enrolled. Among the whole group, 72 (13.9%) patients had pathogenic or likely pathogenic *BRCA1* (*n* = 24, 4.6%) or *BRCA2* (*n* = 48, 9.3%) mutations, while 53 (10.3%) others had VUS. Among 333 younger (≤40 years) patients, mutations were observed in 44 (13.2%). Positive mutations were found in 40 (16.5%) patients with one or more close relatives with breast cancer and in 20 (35.1%) of the 57 patients with triple-negative disease. Multivariate analysis showed that a triple-negative status, history of two or more close relatives with breast cancer, and history of one or more close relatives with invasive ovarian cancer were associated with significant high odds ratios (OR) of carrying a pathogenic variant, with an OR (95% CI) of 5.08 (2.66–9.67), 3.24 (1.78–5.89), and 2.97 (1.04–8.52), respectively.

**Conclusions:**

*BRCA1 and BRCA2* mutations are not uncommon among Jordanian patients. Young age has the weakest association with positive mutations, while patients with triple-negative disease, especially those with an additional positive family history, have the highest mutation rate.

## 1. Introduction

Accounting for almost 20% of all cancer cases, breast cancer continues to be the most common cancer and the leading cause of cancer-related deaths among Jordanian women. A total of 1145 cases were reported by the Jordan Cancer Registry in its latest annual report [1]; more than 60% of them are treated at our center. Almost 50% of breast cancer patients are diagnosed at the age of 50 or younger. Regionally, more than a third of patients present with locally-advanced or metastatic disease [2, 3].

Published data had shown that 5–10% of breast cancer is hereditary and mostly related to *BRCA1* or *BRCA2* gene mutations [4, 5]. Efforts to identify such mutations are extremely important given the high penetrance rates among its carriers [6]. In a meta-analysis of published studies, the estimated mean cumulative risk for breast and ovarian cancers by 70 years of age for *BRCA1* mutation carriers were 57% and 40%, respectively, while carriers of *BRCA2* mutation had a risk of 49% and 18%, respectively [7]. Risk-reduction interventions, such as bilateral mastectomies and oophorectomies, are highly recommended in such patients. More recently, data had shown that specific breast cancer treatment may be informed by the *BRCA1* or *BRCA2* mutation status. In patients with advanced breast cancer associated with *BRCA1* or *BRCA2* mutations, olaparib and talazoparib are now approved for treatment [8–11].

Data related to hereditary breast cancer among the Arab countries in general, and Jordan in particular, are scarce, and ranges of positive rates are very wide [12–18]. Knowledge about the pattern and prevalence of ancestry-specific prevalence of *BRCA1* and *BRCA2* mutations can help policy makers tailor counselling, prevention, and treatment strategies that can better help our particular patients. We recently reported our experience with 100 high-risk patients (median age: 40, range: 22–75 years) treated and followed at our institution. In total, 20 (20.0%) patients had deleterious (pathogenic) and 7 (7.0%) others had suspected deleterious (likely pathogenic) mutations in *BRCA1* or *BRCA2* genes. Highest mutation rates were observed among patients with triple-negative disease (negative for estrogen receptors (ER), progesterone receptors (PR), and human epidermal growth factor (HER2) receptors), especially among those with a positive family history of breast and/or ovarian cancer, patients with bilateral or second primary breast cancer, and those with a family history of male breast cancer [19].

The aim of our study is to evaluate, in a larger group of patients, the contribution of germline mutations in *BRCA1* and *BRCA2* to breast cancer among Jordanian patients with selected high-risk profile as per the National Comprehensive Cancer Network (NCCN) guidelines [20].

## 2. Methods

Jordanian breast cancer patients with selected high-risk profile, as per the NCCN guidelines [20], were invited for *BRCA1* and *BRCA2* testing. This includes patients of 40 years of age or younger at the time of breast cancer diagnosis, patients with at least two breast cancer primaries (i.e., bilateral tumors or 2 or more clearly separate ipsilateral tumors, occurring synchronously or asynchronously), the first at the age of 50 years or younger, patients diagnosed at the age of 50 years or younger with one or more close relatives with breast cancer at any age, diagnosed at any age with 2 or more close relatives with breast cancer at any age, diagnosed at any age with one or more close relatives with invasive ovarian cancer diagnosed at any age, diagnosed at any age with a close male relative with breast cancer at any age, and patients with triple-negative disease who are 60 years of age or younger [20]. All patients had their diagnosis, treatment, and follow-up at our center.

Eligible patients were identified by their primary oncologists during their routine clinic visit or during the weekly breast multidisciplinary team (MDT) discussion. Patients who consented to *BRCA1* and *BRCA2* testing were then referred to our genetic counseling clinic where a lengthy interview and discussion by a trained genetic counselor were carried out. Clinical and psychosocial consequences of positive test results were discussed at length with the patients, and when requested by the patient, such meeting and discussion were also carried out with the spouse and/or family members.

The study was discussed and approved by our Institutional Review Board (IRB), and all patients signed informed consent. *BRCA1* and *BRCA2* testing were performed at no cost to participants as per part of the routine clinical practice. Ten milliliters of peripheral blood samples were obtained for DNA extraction. *BRCA1* and *BRCA2* sequencing were performed at Leeds Cancer Center, Leeds, United Kingdom. Based on a standardized variant assessment tool used by reference genetics labs, *BRCA1* and *BRCA2* mutations were classified as pathogenic/likely pathogenic (positive) and variant of uncertain significance (VUS). Clinical and pathological data were obtained from patients' medical records, and a detailed 3-generation family history was also obtained by a genetic counselor or one of the investigators.

Analysis was performed using an Agilent SureSelect custom design reagent to screen for germline pathogenic variants. Genomic DNA regions including coding exons and intron/exon boundaries are targeted by hybridization capture and sequenced on the Illumina platform with a sensitivity of at least 95%. The target region of selected transcripts is covered to a minimum read depth of 30x. Analysis for large deletion and duplication is preformed using comparative depth of coverage of NGS data and/or MLPA analysis using P087, P045, and P260.

### 2.1. Statistical Analysis

Patient characteristics were tabulated and described by ranges, medians, or percentages (%). First-degree close relatives diagnosed with breast cancer and tested later to the index case in the family were excluded from analyses. The *χ*2 test or Fisher exact test were used to compare the proportion of positive *BRCA1* and *BRCA2* pathogenic/likely pathogenic mutations according to the age (≤40 versus >40), triple-negative status, first- and/or second-degree family history of breast and/or ovarian cancer, bilateral or second primary breast cancer, number of indications for genetic testing, and family history of male breast cancer. Multivariate analysis using a logistic regression model adjusting for the age, triple-negative status, and bilateral or second primary breast cancer was performed. Odds ratios and their related 95% confidence intervals (CI) were calculated. A *P* value ≤ 0.05 was considered significant. Version 9.4 of SAS software (SAS Institute Inc., Cary, NC) was utilized.

## 3. Results

Between November 2016 and January 2019, a total of 517 consecutive eligible patients were recruited. The median age of participants was 39 (range: 19–78) years. At the time of diagnosis, 333 (64.4%) patients were 40 years of age or younger. Majority (*n* = 420, 81.2%) of the patients had hormone receptor (ER and/or PR) positive disease. Human epidermal growth factor receptor (HER2) was positive in 133 (25.7%) by immunohistochemistry (IHC) and/or Fluorescent In Situ Hybridization (FISH), and 57 (11.0%) had triple-negative disease, Table 1.

Among the whole group, 72 (13.9%) patients had pathogenic/likely pathogenic *BRCA1* or *BRCA2* mutations; 48 (66.7%) of them were in *BRCA2*, while 24 (33.3%) in *BRCA1* and an additional 53 (10.3%) patients had VUS. A total of 242 (46.8%) had their genetic testing because they were 50 years of age or younger with one or more close relatives with breast cancer at any age; 40 (16.5%) of them were positive for *BRCA1* or *BRCA2*. Among the 333 patients who were 40 years of age or younger, the pathogenic/likely pathogenic mutations were observed in 44 (13.2%) patients, Table 2.

**Figure 1:**
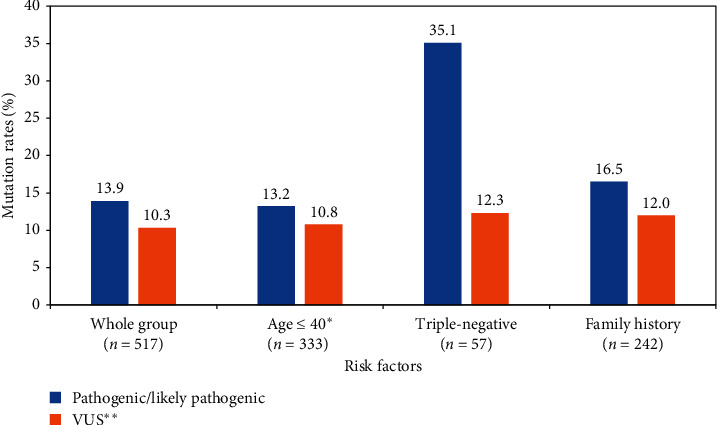
*BRCA1*and *BRCA2* mutation rates by indication.

**Figure 2:**
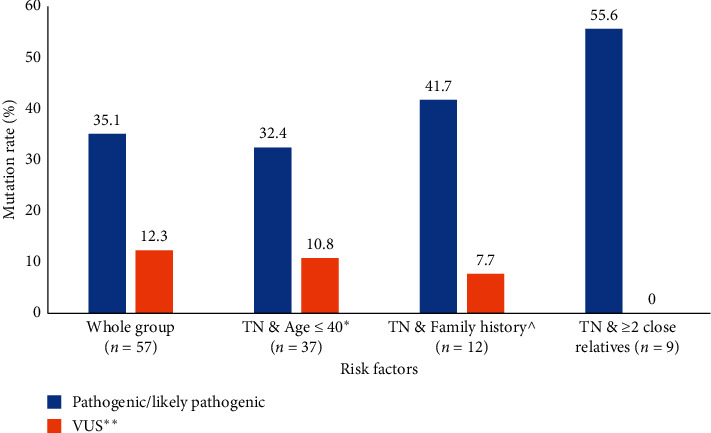
*BRCA1*and *BRCA2* mutations among patients with triple-negatives.

Twenty (35.1%) of the 57 patients with triple-negative disease had pathogenic/likely pathogenic mutations; 16 (80.0%) of them were in *BRCA1*, and only 4 (20.0%) were in *BRCA2*. An additional 7 (12.3%) others had VUS. Among 37 patients with triple-negative disease who were 40 years of age or younger, 12 (32.4%) were positive; all except 2 were in *BRCA1*. Among the patients with triple-negative disease who have a family history of breast cancer diagnosed at an age <50 (*n* = 12), 5 (41.7%) were *BRCA1*- or *BRCA2*-positive. Another 5 (55.6%) of those with two family members with breast cancer at any age (*n* = 9) were positive for pathogenic mutation too.  Figure 1 illustrates the mutation rates among patients tested for different indications, while Figure 2 details mutation rates among subgroups of patients with triple-negative disease.

We also reviewed the mutation rates based on the number of indications a patient may have had for testing. Among 205 (39.7%) patients who had only one indication as per the NCCN guidelines, only 12 (5.9%) had pathogenic or likely pathogenic mutation compared to 25 (15.1%) among 166 (32.1%) patients with two indications and 35 (24.0%) among 146 (28.2%) patients with three or more indications. No founder mutation was identified, and the type and frequency of specific mutations are illustrated in Table 3.

Multivariate analysis using a logistic regression model was performed. The triple-negative status, history of two or more close relatives with breast cancer, and history of one or more close relatives with invasive ovarian cancer were significant with an OR of carrying a pathogenic variant (95% CI) of 5.08 (2.66–9.67), 3.24 (1.78–5.89), and 2.97 (1.04–8.52), respectively (Table 4).

## 4. Discussion

This is the biggest *BRCA1* and *BRCA2* mutation study from Jordan and one of the biggest from the region. Our data showed that such mutations are not uncommon among Jordanian patients selected and tested as per the NCCN guidelines. Contrary to what is usually seen in western societies, our data indicate that mutation rates are not higher among the younger patients (13.2%) compared to the whole group (13.9%), obviously all with at least one risk factor. The fact that breast cancer is diagnosed at a younger age in our region can be a factor. We are in the process of combining the cohort of younger patients included in this study and our previous one [19] to study the contribution of age, in the absence of other risk factors, to the risk of carrying *BRCA1* or *BRCA2* mutation.

We also noted relatively high rates of VUS in this group of patients. This could likely be related to racial issues as western reference laboratories might not have enough exposure to the specific mutations encountered in our population. Mutation testing in our previously published study [19] was conducted at a different reference laboratory and had a similar high VUS rate, making the racial hypothesis an interesting one to follow.

Patients with triple-negative disease had the highest mutation rate and, as expected, mostly (80%) in *BRCA1*. Given this high positive pathogenic mutation rate, additional risk factors did not add to the already high mutation rate. The only exception is probably the presence of a family history of breast cancer in at least two family members. However, studies including a larger number of such patients are needed to address this issue. In addition to patients with triple-negative disease, patients with at least two breast primaries had higher mutation rate (21.7%).

Our positive mutation rates are significantly higher than what our colleagues had recently reported among 281 Lebanese patients [21]. Though it was stated that patients were tested as per NCCN guidelines for mutation screening, the prevalence of mutated *BRCA1* or *BRCA2* genes was only 6.0% and 1.4%, respectively. In an earlier study, reported by the same group, on 250 Lebanese patients tested between 2009 and 2012 who were considered to be at high risk of carrying *BRCA1* or *BRCA2* mutations because of presentation at a young age and/or a positive family history of breast or ovarian cancer, 14 (5.6%) carried a deleterious mutation (7 *BRCA1*, 7 *BRCA2*) and 31 (12.4%) carried VUS. In the same study, only one (1.4%) of the 74 patients aged ≤40 years without a family history had pathogenic mutation, while 8 (10.8%) of the 74 patients aged ≤40 years with a positive family history had a deleterious mutation [22]. On the other hand, a recent systematic review and meta-analysis of 14 studies from the region attempted to better describe the prevalence of *BRCA1* and *BRCA2* mutations in Arab countries. The study has several methodology problems, yet they reported a high rate of 20%. [23].

We have no explanation on the significant differences between our rates and what had been reported among the Lebanese. There should be no significant ethnic differences that may account for such variation. In our database, 63 non-Jordanian patients from Syria, Iraq, Libya, and Palestine and not included in our analysis were tested for *BRCA1* and *BRCA2* mutation following the same indications and methodology of testing, and 8 (12.7%) had positive mutations. Different inclusion criteria for testing or different testing methodologies may have contributed to this variation in mutation rates.

In our previous study, we addressed the challenges in conducting such studies in a culturally sensitive society with limited resources. Many ethical and cultural difficulties were encountered and continued to be encountered during the course of our study. Ensuring confidentiality and privacy are still major issues in a closely related, relatively small community. However, very few patients (4 patients) expressed their concerns about labeling and stigmatization and, thus, refused the testing when approached by their physicians. Additionally, none of the patients tested positive had issues with sharing and addressing such results with their at-risk family members. Local or regional data on clinical and psychosocial consequences related to positive mutations are lacking. We are in the process of collecting such data as part of a larger genetic testing and genetic counselling project.

Potential employment and social discrimination addressed in our previous report had not surfaced out as major issues in expanding our program. However, insurance issues continued to be a problem. Though governmental cancer care insurance covers for *BRCA1* and *BRCA2* testing, it does not cover the major part of the reconstruction surgery.

Now that we confirm that *BRCA1* and *BRCA2* mutations are not uncommon, such testing, counseling, and linking it to risk-reduction surgery should be incorporated into the routine clinical practice nationwide. At our institution, genetic testing has become routinely offered for at-risk patients as per the published NCCN guidelines. Additionally, a clinical cancer genetics program was established and operating smoothly with no major issues. Compliance on testing high-risk patients was recently added to our “Key Performance Indicators (KPI),” data on which are collected and reported by our quality office. We are also currently expanding our genetic testing and counseling program to include mutations in genes other than *BRCA1* and *BRCA2* for high-risk patients who were tested negative.

The current study has avoided many of the limitations we had in the previous study. The sample size is not an issue here, though larger studies are needed to study the contribution of each risk factor in its own or in combination to a positive mutation rate.

## 5. Conclusions

Our recent findings support the conclusion that *BRCA1* and *BRCA2* mutations are prevalent enough to be incorporated into clinical practice guidelines nationwide and to provide affected women with free access to risk reduction and reconstructive surgeries.

## Figures and Tables

**Table 1:** Patient characteristics (*n* = 517).

Characteristics		Number	(%)
Age at diagnosis (years)	Median	39	
	Range	19–78	

Hormonal status	ER-positive	392	75.8
	PR-positive	375	72.5
	ER- or PR-positive	420	81.2
	ER- and PR-negative	97	18.8

HER2 status	HER2-positive	133	25.7
	HER2-negative	318	61.5
	Unknown	66	12.7

Triple-negative		57	11.0

Positive family history of breast cancer		441	85.3

ER: rstrogen receptors; PR: progesterone receptors; HER2: human epidermal growth factor receptor.

**Table 2:** Rates of positive *BRCA1* and *BRCA2* mutation across different indications.

Variable		Total	Positive mutations			
			*BRCA1*	*BRCA2*	*BRCA1* and *BRCA2*	*P* value^*∗*^
Age at diagnosis (years)	≤40	333	15 (4.5%)	29 (8.7%)	44 (13.2%)	0.530
	>40	184	9 (4.9%)	19 (10.3%)	28 (15.2%)	

Age ≤ 50 years with one or more close relatives with breast cancer at any age	Yes	242	8 (3.3%)	32 (13.2%)	40 (16.5%)	0.1
	No	275	16 (5.8%)	16 (5.8%)	32 (11.6%)	

Age ≤ 60 with triple-negative disease	Yes	57	16 (28.1%)	4 (7.0%)	19 (35.1%)	<0.001
	No	460	8 (1.7%)	44 (9.6%)	53 (11.5%)	

Any age with at least 2 breast cancer primaries	Yes	57	4 (7.0%)	4 (7.0%)	8 (14.0%)	0.98
	No	460	20 (4.3%)	44 (9.6%)	64 (13.9%)	

Any age with 2 or more close relatives with breast cancer	Yes	115	7 (6.1%)	21 (18.3%)	28 (24.3%)	<0.001
	No	402	17 (4.2%)	27 (6.7%)	44 (10.9%)	

Any age with one or more close relatives with invasive ovarian cancer diagnosed at any age	Yes	19	2 (10.5%)	4 (21.1%)	6 (31.6%)	0.023
	No	498	22 (4.4%)	44 (8.8%)	66 (13.3%)	

All patients		517	24 (4.6%)	48 (9.3%)	72 (13.9%)	

^*∗*^ *P* value comparing risk factor categories.

**Table 3:** Frequency of *BRCA1* and *BRCA2* mutations.

Gene	Exon/intron	Nucleotide change	Amino acid change	Variant type	dbSNP rs	Clinical significance	Database report	Frequency (*n*)
*BRCA1*	Exon 2	c.66dup	p.Glu23Arg	Duplication/fs	rs80357783	Pathogenic	Yes	5
*BRCA1*	Exon 12	c.4117G > T	p.Glu1373Ter	Nonsense	rs80357259	Pathogenic	Yes	3
*BRCA1*	Intron 17	c.5074 + 3A > G	Splice acceptor	Intervening sequence	rs80358181	Likely pathogenic	Yes	3
*BRCA1*	Exon 11	c.4065_4068del	p.Asn1355Lys	Deletion/fs	rs80357508	Pathogenic	Yes	2
*BRCA1*	Exon 18	c.5123C > A	p.Ala1708Glu	Missense	rs28897696	Pathogenic	Yes	2
*BRCA1*	Exon 3	c.121C > T	p.His41Tyr	Missense	rs1060502353	Likely pathogenic	Yes	2
*BRCA2*	Exon 11	c.2254_2257 del	p.Asp752Phefs	Deletion/fs	rs80359326	Pathogenic	Yes	8
*BRCA2*	Exon 11/Exon 11	c.2254_2257 del & c.5351 dup	p.Asp752Phefs and p.Asn1784Lysfs	Deletion/fs-Duplication/fs	rs80359326 & rs80359508	Pathogenic	Yes	6
*BRCA2*	Exons 5-11	Partial duplication (exons 5-11)	Absent or disrupted protein product	Large duplication	*—*	Pathogenic	Yes	5
*BRCA2*	Exon 10	c.1233dup	p.Pro412Thr	Duplication/fs	rs80359270	Pathogenic	Yes	3
*BRCA2*	Exon 11	c.6685G > T	p.Glu2229Ter	Nonsense	rs730881548	Pathogenic	Yes	3
*BRCA2*	Exon 11	c.6486_6489del	p.Lys2162Asn	Deletion/fs	rs80359598	Pathogenic	Yes	2
*BRCA2*	Intron 24	c.9257-1G > A	Splice acceptor	Intervening sequence	rs81002889	Likely pathogenic	Yes	2

**Table 4:** Logistic regression.

Variable	Odds ratio	95% CI	*P* value
Age at diagnosis <40	1.27	0.71–2.28	0.40
Triple negative	5.08	2.66–9.67	<0.0001
Bilateral or second primary breast cancer	1.01	0.43–2.36	0.99
History of two or more close relatives with breast cancer	3.24	1.78–5.89	0.0001
History of one or more close relatives with invasive ovarian cancer	2.97	1.04–8.52	0.043

CI : confidence interval.

## Data Availability

Data will not be available online as it might contain sensitive information regarding the mutation status.

## Abbreviations

CI: Confidence intervals
ER: Estrogen receptors
PR: Progesterone receptors
FISH: Fluorescent in situ hybridization
HER2: Human epidermal growth factor receptor
IHC: Immunohistochemistry
IRB: Institutional review board
MDT: Multidisciplinary team
NCCN: National comprehensive cancer network
VUS: Variant of uncertain significance.
